# Impact of sleep chronotype on blood pressure and metabolic markers

**DOI:** 10.3389/fneur.2025.1510222

**Published:** 2025-02-12

**Authors:** Weiwei Huang, Qinglu Wang, Yuan Gao, Jiefeng Wang, Feihao Wang, Jiyou Tang

**Affiliations:** ^1^Department of Neurology, The First Affiliated Hospital of Shandong First Medical University and Shandong Provincial Qianfoshan Hospital, Jinan, China; ^2^Department of Intensive Care Unit, Dezhou Second People's Hospital, Dezhou, China; ^3^Department of Intensive Care Unit, The Second Affiliated Hospital of Shandong First Medical University, Tai'an, China; ^4^Department of Neurology, Tengzhou Central People's Hospital, Zaozhuang, China; ^5^Department of Neurology, Liaocheng People’s Hospital, Liaocheng, China

**Keywords:** sleep chronotype, metabolic markers, Morningness-Eveningness Questionnaire, blood pressure, creatinine, aspartate aminotransferase

## Abstract

**Objective:**

The aim of this study was to investigate the correlation between sleep chronotype and metabolic markers to further reveal the influence of sleep chronotype on human health.

**Methods:**

The Morningness-Eveningness Questionnaire was administered to 442 volunteers aged 23–70 years old. The sleep chronotype was divided into morning type (167 cases), neither type (224 cases), and evening type (51 cases). Blood pressure was recorded, and fasting venous blood samples were collected to assess liver function, renal function, blood glucose levels, blood lipid profile, and other biochemical parameters.

**Results:**

1. There was a statistically significant difference in the age of the morning type, neither type, and evening type (*p* < 0.01), but there was no significant difference in gender, height, weight, and BMI (*p*s > 0.05). 2. After controlling for the influence of age-related factors, significant differences were seen between morning type, neither type, and evening type groups in creatinine and aspartate aminotransferase (*p* < 0.05). 3. After controlling for the influence of age-related factors, the evening type group had lower systolic blood pressure, diastolic pressure, and mean arterial pressure (MAP) compared to the morning type and neither type (*p* < 0.05). 4. After controlling the influence of age-related factors, the MEQ scores were positively correlated with systolic blood pressure, diastolic blood pressure, and mean arterial pressure (*r* = 0.099, 0.096, 0.104, *p*s < 0.05).

**Conclusion:**

The evening sleep type is more prone to daytime sleepiness. There were significant differences observed in systolic blood pressure, diastolic blood pressure, and MAP. These variations may be linked to the effects of different sleep chronotype on blood pressure regulation or the blood pressure’s autonomous rhythm.

## 1 Introduction

Sleep chronotype can be categorized into morning type, evening type, and neither type. The Morningness-Eveningness Questionnaire (MEQ) is currently the most widely used scale for assessing sleep chronotype. Research indicates that its effectiveness in evaluating circadian rhythm types is comparable to that of actigraphy (1). Horne and Östberg developed and first applied the Morningness-Eveningness Scale with 19 items (MEQ-19) in 1976 (2). The MEQ has been translated into multiple languages in various countries and regions and has been applied to determine sleep chronotype, all of which have undergone reliability and validity testing (3–5). Different sleep chronotype can affect both physical and mental health. Research shows that evening type exhibit more unhealthy dietary habits and behavioral health issues, making them more susceptible to cardiovascular diseases, microvascular events, and metabolic disorders such as type 2 diabetes (6, 7). Other studies have also indicated that sleep chronotype is associated with depressive symptoms, with evening type having a higher likelihood of experiencing depression (8).

### 1.1 Sleep chronotype plays an important role in metabolic diseases

Metabolic syndrome (MS) is a condition characterized by a cluster of central obesity, hyperglycemia, elevated blood pressure, and dyslipidemia, which are risk factors for cardiovascular diseases. Studies suggest that morning type may be more closely related to obesity and high blood pressure (9). In a health study conducted within a Hispanic community, an association was found between the neither type population aged over 40 and a higher risk of metabolic syndrome, while evening type were significantly associated with a lower risk of metabolic syndrome among participants under 40 years of age (10). Additionally, research has shown that evening type are associated with metabolic syndrome in females, whereas males do not exhibit this association (11). Metabolic dysfunction associated fatty liver disease (MASLD) refers to the accumulation of excessive triglycerides in the liver, resulting in hepatic steatosis, also known as isolated fatty liver (ILS), which is the basic characteristic of MASLD. MAFLD is a more appropriate overarching term used to describe liver diseases associated with metabolic dysfunction rather than nonalcoholic fatty liver disease (NAFLD) (12). A review analyzed the role of circadian rhythm in NAFLD, which examined the effects of genetic background, hormone homeostasis, gut microbiota, and sleep habits. NAFLD is more common among daytime nap takers and shows a dose-dependent relationship with nap duration (13). An increase in NAFLD frequency has also been observed in patients who frequently eat dinner (14). So far, no clear association has been found between shift work and an increase in NAFLD incidence. Another study found that there were no statistically significant differences in sleep quality, nighttime dietary habits, or temporal preferences among patients with varying degrees of MASLD (15). The circadian rhythm also plays an important role in regulating lipid metabolism and hormonal homeostasis. The circadian rhythm affects lipid synthesis, absorption, and transport, this may be related to the regulation of Clock gene and PER2 gene (16). In humans, induced sleep wake misalignment leads to unscheduled secretion of insulin, leptin, and norepinephrine, while cortisol, adrenaline, and glucose maintain a normal circadian rhythm secretion pattern (17). However, there is no research on the correlation between sleep chronotype and metabolic diseases in China.

Therefore, this study explores the correlation between sleep chronotype and metabolic markers, revealing differences in physiological and biochemical indicators among different sleep types, and further elucidates the impact of sleep chronotype on human health, providing a theoretical foundation for promoting healthy sleep.

## 2 Materials and methods

### 2.1 Subjects

From September 2019 to December 2023, a total of 442 volunteers aged 20–65 who had been living in Jinan, Shandong Province for an extended period participated in this study. The research protocol has been approved by Shandong Qianfoshan Hospital, and the study plan was explained to each participant, who provided written informed consent. All participants were Han ethnicity. Inclusion criteria were: recent health examination reports indicating good health; mental health with a Pittsburgh Sleep Quality Index (PSQI) score ≤ 5 (18); the ability to understand and cooperate, had no additional gym or outdoor exercise in the past 2 months, daily exercise forms are walking or yoga, with an average daily exercise duration of about 40–60 min. Exclusion criteria included: athletes, shift and rotating workers; those who frequently traveled long distances or frequently adjusted to time zone changes; individuals suffering from insomnia or other sleep disorders such as sleep apnea or periodic limb movement disorder; those with severe physical diseases like heart disease, tumors, blood diseases, thyroid diseases, severe injuries and infections, chronic pain, and mental disorders such as anxiety and depression; and the use of medications that impact sleep, such as hypnotics and antipsychotics. Volunteers completed the PSQI and Epworth Sleepiness Scale (ESS) assessments in the sleep center.

### 2.2 Morningness-Eveningness Questionnaire (MEQ)

The MEQ consists of 19 self-assessment items, with total scores ranging from 16 to 86. The questionnaire mainly assesses an individual’s sleep–wake times, optimal times for physical and cognitive activities, subjective alertness upon waking, and the times of day when fatigue is greatest. Out of the 19 items, 11 items are scored from 1 to 4 points, 2 items from 0, 2, 4, to 6 points, 1 item from 0, 2, 3, to 5 points, and the remaining 5 items are scored from 1 to 5 points. The total score is obtained by summing the scores of each item. The scoring criteria for the five types are as follows: 70–86 points for absolute morning type; 59–69 points for moderate morning type; 42–58 points for neither type; 31–41 points for moderate evening type; and 16–30 points for absolute evening type (2). In this study, absolute morning type and moderate morning type are collectively referred to as morning type, and absolute evening type and moderate evening type are collectively referred to as evening type. Based on the MEQ, subjects were categorized into morning type, intermediate type, and evening type.

### 2.3 Blood pressure and biochemical indices measurement

All subjects underwent venipuncture in a fasting state at 8 AM and had their blood pressure measured after sitting for 20 min. Blood specimens were sent to the Department of Laboratory Medicine at Qianfoshan Hospital (using a Bayer 2,400 fully automated biochemical analyzer) to measure biochemical indices including alanine aminotransferase (ALT), aspartate aminotransferase (AST), glomerular filtration rate (GFR), uric acid, glucose, triglycerides, total cholesterol, high-density lipoprotein (HDL), low-density lipoprotein (LDL), serum creatinine, and uric acid. All biochemical specimens were analyzed on the same day, conducted by an experienced laboratory technician and reviewed by a senior laboratory physician who provided the test results.

### 2.4 Statistical analysis

Statistical analysis was performed using IBM SPSS Statistics 25. The normality of the data was first tested. Normally distributed continuous data were expressed as mean ± standard deviation (±s). Since age influenced scores on the MEQ, as well as physiological and biochemical indices, covariance analysis (adjusting for age factors) was used to compare the three groups of morning type, neither type, and evening type. Chi-square tests were used to compare count data among the three groups, and partial correlation analysis was conducted for correlation analysis of the data. A *p*-value <0.05 was considered statistically significant.

## 3 Result

### 3.1 Comparison of demographic data among morning type, neither type, and evening type individuals

A total of 442 volunteers participated in this study: 167 morning type (130 males, 37 females), with an average age of (51.90 ± 10.04) years; 224 neither type (187 males, 37 females), with an average age of (46.89 ± 10.21) years; and 51 evening type (42 males, 9 females), with an average age of (42.59 ± 9.55) years. There were no statistically significant differences in terms of gender (χ^2^ = 2.042, *p* = 0.360), height (*F* = 0.880, *p* = 0.416), weight (*F* = 0.478, *p* = 0.620), and BMI (*F* = 0.350, *p* = 0.705) among the three groups; however, the age decreased successively among the morning type, neither type, and evening type groups (*F* = 21.068, *p* = 0.000), with significant statistical significance (Table 1).

**Table 1:** Comparison of demographic characteristics among morning type, neither type, and evening type.

	Morning type (*n* = 167)	Neither type (*n* = 224)	Evening type (*n* = 51)	*p* value
Male/female	130/37	187/37	42/9	0.360
Age (years)	51.90 ± 10.04	46.89 ± 10.21	42.59 ± 9.55	0.000*
Height (cm)	170.25 ± 7.98	172.27 ± 7.33	172.88 ± 7.94	0.416
Weight (kg)	75.43 ± 11.74	77.30 ± 11.70	76.70 ± 10.46	0.620
BMI	25.91 ± 2.83	25.96 ± 2.94	25.65 ± 2.96	0.705

*: *p* < 0.05.

### 3.2 Comparison of sleep factors among morning type, neither type, and evening type individuals

After controlling for age, there were no statistically significant differences in total sleep time (*F* = 2.694, *p* = 0.069) or sleep efficiency (*F* = 0.904, *p* = 0.406) among the morning type, neither type, and evening type groups. However, there were significant differences in ESS scores (*F* = 4.926, *p* = 0.008), with evening type individuals being more likely to experience daytime sleepiness (Table 2).

**Table 2:** Comparison of Sleep factors among morning type, neither type, and evening type.

	Morning type (*n* = 167)	Neither type (*n* = 224)	Evening type (*n* = 51)	*p* value
Total sleep time (h)	7.36 ± 1.25	7.11 ± 0.97	6.96 ± 0.95	0.069
Sleep efficiency (%)	89.31 ± 10.98	89.09 ± 9.75	87.43 ± 9.09	0.406
ESS score	5.95 ± 3.35	6.76 ± 3.73	7.91 ± 4.03	0.008*

*: *p* < 0.05. ESS: Epworth Sleepiness Scale.

### 3.3 Comparison of metabolic markers among morning type, neither type, and evening type individuals

**Figure 1:**
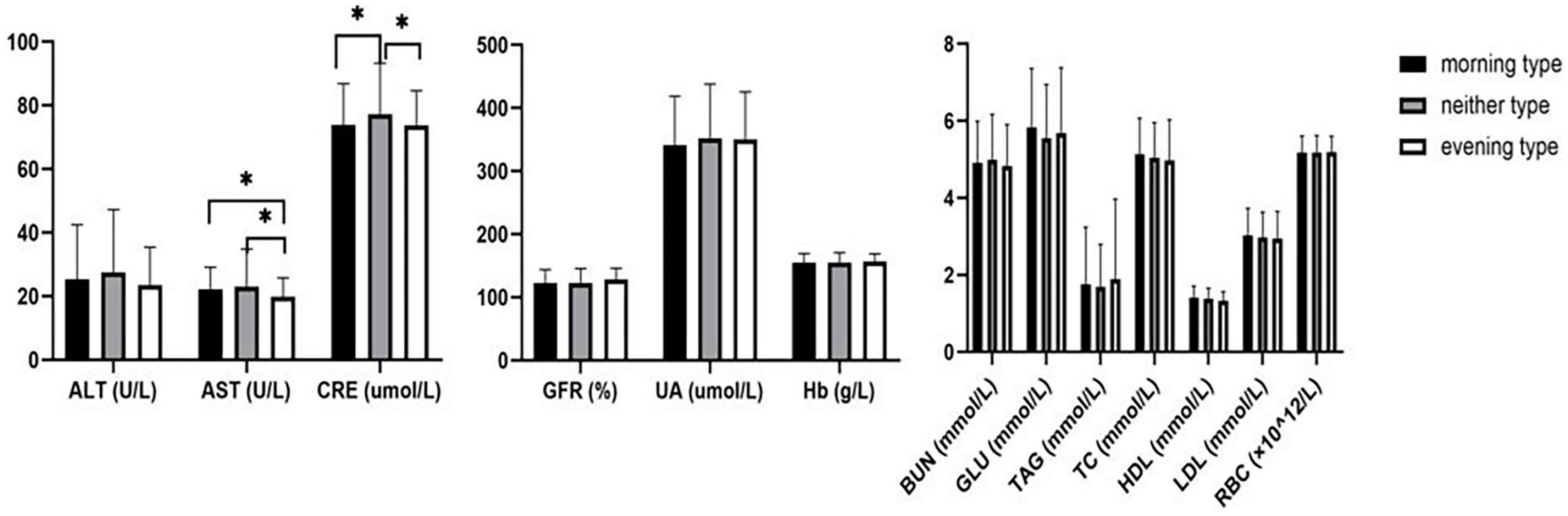
Comparison of biochemical indexes among morning type, neither type, and evening type. *: *p* < 0.05. ALT: alanine aminotransferase; AST: aspartate aminotransferase, BUN: blood urea nitrogen; CRE: creatinine; UA: uric acid; TAG: triglyceride; TC: total cholesterol; HDL: high-density lipoprotein; LDL: low-density lipoprotein; GLU: glucose; GFR: glomerular filtration rate; RBC: red blood cell; Hb: hemoglobin.

After controlling for age, the average levels of AST among morning type and neither type individuals were similar and slightly higher than those of evening type individuals (*F* = 3.119, *p* = 0.045). The average serum creatinine levels of morning type and evening type individuals were close and higher than that of neither type individuals (*F* = 3.336, *p* = 0.036), showing statistically significant differences. No statistically significant differences were found in other biochemical indicators, including ALT (*F* = 2.198, *p* = 0.112), glomerular filtration rate (*F* = 1.890, *p* = 0.152), uric acid (*F* = 0.821, *p* = 0.441), glucose (*F* = 0.901, *p* = 0.407), triglycerides (*F* = 0.443, *p* = 0.642), total cholesterol (*F* = 0.106, *p* = 0.899), HDL (*F* = 0.969, *p* = 0.380), LDL (*F* = 0.118, *p* = 0.889), red blood cell count (*F* = 0.905, *p* = 0.405), and hemoglobin content (*F* = 0.205, *p* = 0.815) (Figure 1).

### 3.4 Comparison of blood pressure among morning type, neither type, and evening type individuals

**Figure 2:**
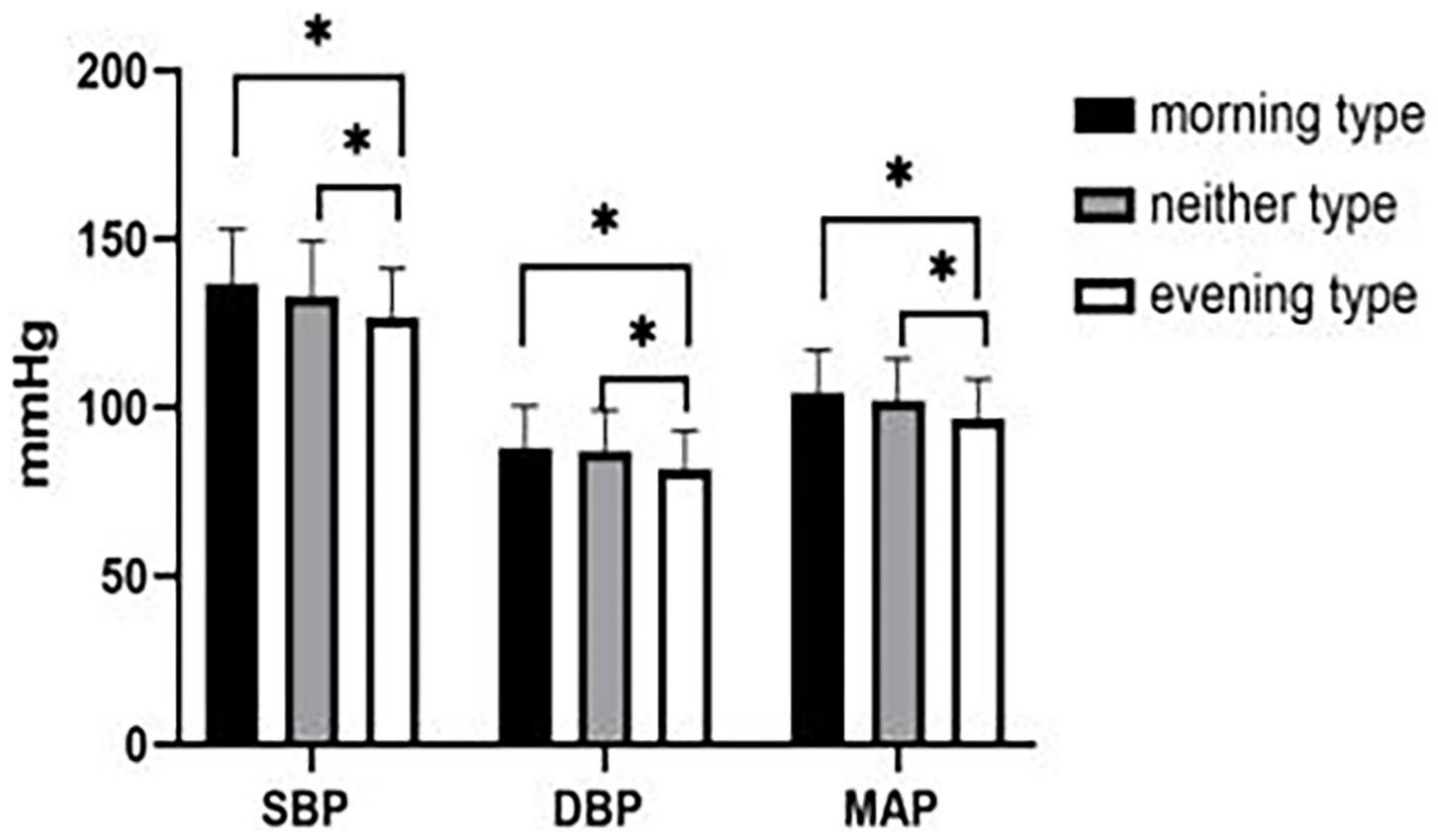
Comparison of blood pressure among morning type, neither type and evening type. *: *p* < 0.05. SBP: systolic blood pressure, DBP: diastolic blood pressure, MAP: mean arterial pressure.

After controlling for age, systolic blood pressure (SBP) (*F* = 3.992, *p* = 0.019), diastolic blood pressure (DBP) (*F* = 5.155, *p* = 0.006), and mean arterial pressure (MAP) (*F* = 5.414, *p* = 0.005) of morning type, neither type, and evening type decreased in turn, and the differences were statistically significant (Figure 2).

### 3.5 Correlation analysis between MEQ scores and blood pressure

After controlling for age, MEQ scale scores were positively correlated with systolic blood pressure (*r* = 0.099, *p* = 0.038), diastolic blood pressure (*r* = 0.096, *p* = 0.044), and mean arterial pressure (*r* = 0.104, *p* = 0.029) (Table 3).

**Table 3:** Correlation analysis between MEQ scores and blood pressure.

	MEQ (score)	r value	*P* value
SBP (mmHg)	0.099	0.099	0.038*
DBP(mmHg)	0.096	0.096	0.044*
MAP (mmHg)	0.104	0.104	0.029*

*: *p* < 0.05. SBP: systolic blood pressure, DBP: diastolic blood pressure, MAP: mean arterial pressure.

## 4 Discussion

In recent years, with the rapid development of modern society and the enrichment of nightlife, the importance of sleep chronotype has gained increasing attention. Sleep chronotype may lead to physiological and psychological changes, ultimately affecting work, study, and daily life. Therefore, exploring the effects of different sleep chronotype on metabolic syndrome is crucial, while also providing a theoretical basis for better guidance on sleep hygiene.

### 4.1 Sleep chronotype and gender, age, BMI

Our research results indicate that there is no significant difference in gender among the three groups; however, age differences were noted. Older individuals tend to have a morning chronotype, whereas younger individuals are inclined towards an evening chronotype, which aligns with previous research findings (19), this study found that with increasing age, the proportion of morning chronotype rises, possibly due to hormonal changes and objective environmental factors. After adulthood, due to work or lifestyle needs, individuals are required to wake up early in the morning and sleep earlier in the evening to ensure sufficient sleep and optimal daytime functionality. The relationship between sleep chronotype in middle-aged and older adults and gender has been debated. Some studies suggest that men over 40 are more likely to be early risers compared to women, which may be partly related to the increasing prevalence of insomnia among women (20). However, other research indicates that women’s circadian rhythm patterns shift more towards morning types after reaching middle age (19). In summary, age has a more significant influence on sleep chronotype than gender. BMI is a commonly used indicator for assessing obesity levels and overall health in populations. Research shows that reduced sleep duration can increase the release of orexin and related hormones (21). Orexin not only regulates food intake but also plays a crucial role in sleep–wake regulation. Increased levels of orexin can lead to increased food intake and reduced energy expenditure, causing obesity; its role in circadian rhythm regulation may also contribute to insomnia (22). Our study found no significant difference in BMI among individuals with different sleep chronotype, likely because the difference of total sleep time between morning and evening types is relatively small. In our future research, we will continue to investigate the differences in orexin levels among different sleep chronotype, aiming to reveal its impact on population obesity and health.

### 4.2 Sleep chronotype and blood pressure

Research indicates that sleep chronotype affect sleep structure; morning type are more prone to sleep structure disturbances, leading to elevated blood pressure compared to evening type (23, 24). Circadian rhythm disturbances can increase the release of inflammatory factors, impair endothelial function, and lead to increased blood pressure (25). One study found that evening type have a higher risk of cardiac metabolism compared to the other two types (26). However, in another study on shift workers, chronotype were not linked to any metabolic risk factors (27). Our results show that, among the three groups, evening type had the lowest levels of systolic pressure, diastolic pressure, and mean arterial pressure. There is also a study showing that evening type affects BP and HRV, evening type has lower daytime BP than morning type, which is consistent with our research findings (28). Heart rate variability (HRV) reflects the function of the autonomic nervous system (ANS), which means that sleep chronotype may alter blood pressure through autonomic nervous system (28).

### 4.3 Sleep chronotype and metabolic syndrome

Few studies have been published on the relationship between sleep chronotype and metabolic syndrome. One cohort study found a correlation between evening type and metabolic syndrome in women (11). A study in Japan indicated that among evening type patients with type 2 diabetes, alanine aminotransferase and blood glucose levels were higher than those in morning type patients, while high-density lipoprotein levels were lower (29). Evening type consume more energy daily, possibly due to late-night eating increasing blood sugar levels (29). In contrast, our study findings indicated no significant difference in fasting blood glucose levels among the three groups. This may be because participants were normal and healthy, and the impact of sleep chronotype on metabolic regulation was insufficient to disrupt the self-regulatory mechanisms of blood glucose and blood lipid levels, thus not leading to significant changes.

Our research shows no significant difference in GFR among morning type, neither type, and evening type. However, serum creatinine levels were higher in the neither type compared to morning and evening types, with the mechanisms needing further investigation. Currently, there is no conclusive evidence regarding the relationship between sleep chronotype and renal function. Some studies have found an increased incidence of CKD among night shift workers compared to those with a “healthy sleep” pattern (30). There are also reports indicating that reduced sleep duration can lead to decreased glomerular filtration rate (31), while other scholars found that reduced sleep duration may increase glomerular filtration rate (32). Sleep is a regulator of renal function, inhibiting urine production as well as potassium and sodium excretion during normal sleep. We hypothesize that sleep chronotype may impact nighttime urine production and the excretion of sodium and potassium by regulating the nighttime release of hormones, thereby affecting the variations in glomerular function indicators.

The study revealed that evening type have lower AST levels compared to morning and neither types. There is limited research on the relationship between sleep chronotype and liver function. One study found that non-morning types had a higher incidence of nighttime eating behaviors, but no statistically significant differences in sleep quality, nighttime eating habits, or chronotype among patients with biopsy-proven metabolic dysfunction-associated steatotic liver disease (15). Another study indicated a positive correlation between evening type and metabolic-associated fatty liver disease (33), with further investigation into the mechanisms required.

## 5 Conclusion

Sleep chronotype may influence blood pressure, creatinine level, and AST level, but the specific mechanisms need further confirmation. Therefore, it is particularly important to continue exploring the impact of different sleep chronotype on metabolic syndrome to provide a theoretical basis for better guidance on sleep hygiene. However, this study also has limitations: melatonin is the gold standard for measuring sleep rhythms, and it would be optimal to include sleep data obtained through PSG monitoring (including sleep duration, sleep efficiency, sleep latency, and sleep structure). We did not measure melatonin levels and PSG because it is very complex and costly, but we will conduct a small trial later. We did not measure the morning insulin levels and the impact of social jet lag. In future research, we will use sleep diaries and actigraph to more accurately record social jet lag and its influences, simultaneously pay attention to insulin metabolism.

## Data Availability

The original contributions presented in the study are included in the article/supplementary material, further inquiries can be directed to the corresponding author.
